# Disseminated pustular eruption: Pyoderma gangrenosum as a rare manifestation in antineutrophil cytoplasmic antibody-associated vasculitis

**DOI:** 10.1515/rir-2026-0022

**Published:** 2026-07-13

**Authors:** Mucong Li, Ziqian Wang

**Affiliations:** Department of Rheumatology and Clinical Immunology, Peking Union Medical College Hospital, Chinese Academy of Medical Sciences & Peking Union Medical College, Beijing, China; National Clinical Research Center for Rheumatic and Autoimmune Diseases (NCRC-RAD), National Health Commission, Beijing, China; State Key Laboratory of Complex Severe and Rare Diseases, Peking Union Medical College Hospital, Ministry of Science & Technology, Beijing, China; Key Laboratory of Rheumatology and Clinical Immunology, Ministry of Education, Beijing, China; Beijing Key Laboratory of Intelligence and Precision in Rheumatic and Autoimmune Diseases, Beijing Municipal Commission of Science & Technology, Beijing, China

**Figure 1:**
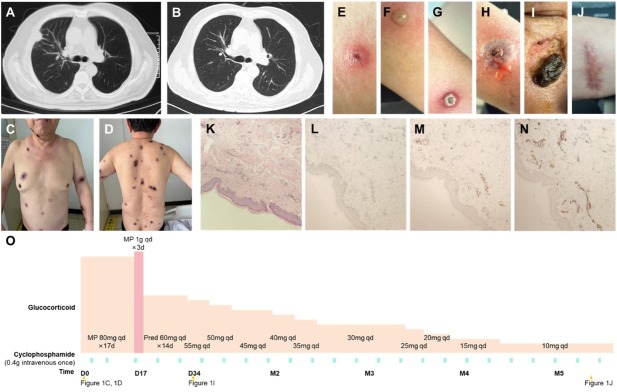
Pulmonary computed tomography (CT) scans, images, pathology and immunohistochemistry of the multiple pustular eruptions. A, Initial pulmonary CT showing a subpleural nodule. B, CT at the onset of pyoderma gangrenosum demonstrating resolution of the nodule. C&D, Multiple pustular eruptions on the head, arms, and trunk upon admission. E-J, Evolution of pyoderma gangrenosum lesions (showing expansion, exudation, crust formation, and subsequent healing with scarring/post-inflammatory changes) after treatment. Skin pathology and immunohistochemistry stained with hematoxylineosin (HE, K), CD20 (L), CD2 (M), and CD31 (N). O, Treatment timeline following the development of pustular eruptions. MP, methylprednisolone.

A 58-year-old man presented with a one-month history of multiple pustular eruptions. He had been diagnosed with antineutrophil cytoplasmic antibody (ANCA)-associated vasculitis (AAV) one year earlier, presenting with persistent cough, intermittent hemoptysis, and fever. Laboratory tests revealed positivity for myeloperoxidase (MPO)-ANCA (172.1 AU/mL) and negativity for anti-glomerular basement membrane antibody and Proteinase 3 (PR3)-ANCA. Pulmonary computed tomography (CT) scan identified bilateral multiple nodules and subpleural nodules (Figure 1A). Treatment with prednisone (60 mg/day) and cyclophosphamide (100 mg/day) was initiated. As symptoms resolved, medications were tapered. Repeat laboratory tests showed decreased MPO-ANCA titer (48.3 AU/mL). Follow-up imaging demonstrated marked regression of the nodules. After ten months of treatment, while on prednisone (7.5 mg every other day) and cyclophosphamide (100 mg/week), the patient self-discontinued all medications due to resolution of symptoms. Fever and cough recurred two weeks later, accompanied by an elevated MPO-ANCA titer (99.8 AU/mL). Pulmonary CT remained stable (Figure 1B). Medium-dose prednisone and mycophenolate mofetil were initiated; however, multiple painless white pustular rashes appeared on both forearms within a month. These lesions gradually enlarged, formed crusts, and disseminated to the head, neck, arms, hands, trunk, scrotum, glan penis, and legs (Figure 1C, 1D, 1E-1J). Bacterial and fungal cultures of pus and blood were negative. Positron emission tomography (PET)/CT showed elevated bone marrow metabolic activity, but bone marrow biopsy revealed no abnormalities. Skin biopsy was consistent with small vessel vasculitis, confirming the diagnosis of pyoderma gangrenosum (Figure 1K-1N). Following glucocorticoid and cyclophosphamide pulse therapy (Figure 1O), the existing lesions gradually healed over five months, with initial signs of crusting and epithelialization observed within two–three weeks of initiating pulse therapy. The MPO-ANCA titer decreased to 24.6 AU/mL.

This case highlights a rare cutaneous manifestation of AAV. While skin involvement occurs in approximately 50% of AAV patients, typically as purpura, segmental edema, nodules, livedo, or urticarial lesions,^[1, 2, 3]^ pyoderma gangrenosum (PG) is reported in less than 1% of cases and has been specifically associated with granulomatosis with polyangiitis (GPA).^[1]^ Unlike the classic ulcerative PG commonly seen in inflammatory bowel disease (IBD) or rheumatoid arthritis (RA), our patient presented with disseminated pustular PG in the context of microscopic polyangiitis (MPA). To our knowledge, this is the first reported case of biopsy-proven, widespread pustular PG in a patient with MPA, characterized by fulminant cutaneous involvement while pulmonary disease remained quiescent, highlighting the heterogeneity of AAV disease activity. Therefore, in such presentations, other common causes of PG, particularly hematologic and solid malignancies, must be excluded.^[4]^ This case underscores that differentiating AAV-associated cutaneous manifestations can be challenging, and biopsy is often essential.
